# What Drives Mortality in *S. maltophilia* Bloodstream Infections? An Outcome-Focused Cohort Study of Treatment Strategies and Resistance Profiles

**DOI:** 10.3390/microorganisms14010077

**Published:** 2025-12-29

**Authors:** Emanuela Zappulo, Nicola Schiano Moriello, Rossella Paolillo, Giulia Zumbo, Biagio Pinchera, Maria Rosaria Catania, Ivan Gentile

**Affiliations:** 1 Infectious Diseases Unit, Department of Clinical Medicine and Surgery, University of Naples Federico II, 80131 Naples, Italy; giuliazumbo@hotmail.com (G.Z.); biagio.pinchera@unina.it (B.P.); ivan.gentile@unina.it (I.G.); 2 Department of Molecular Medicine and Medical Biotechnologies, University of Naples Federico II, 80131 Naples, Italy; rospaolillo@gmail.com (R.P.); mariarosaria.catania@unina.it (M.R.C.)

**Keywords:** *Stenotrophomonas maltophilia*, antimicrobial therapy, treatment duration, microbiological cure, multidrug resistance, Gram-negative bacteria, bacteremia, carbapenem resistance, nosocomial infections, antimicrobial stewardship

## Abstract

*Stenotrophomonas maltophilia* bloodstream infection (SM-BSI) carries high mortality and limited therapeutic options. We conducted a single-center retrospective cohort of adults with first SM-BSI (2018–2024) to describe treatment patterns and identify factors associated with survival. Demographic, clinical, and microbiological data were extracted and analyzed. Forty-three patients were included (median age: 63 years; 61% male). Appropriate antimicrobial therapy was given to 74%; trimethoprim–sulfamethoxazole-based regimens were used in 61%; and combination therapy in 23%. The median time from BSI to treatment initiation was 4 days (IQR: 3–5) and the treatment duration averaged 7 days (IQR: 0–12). Thirty-day mortality was 37% (16/43). The survival analysis found that a 14–21-day course was associated with better 30-day survival than a 7–13-day course (0/9 vs. 5/15 deaths; log-rank *p* = 0.045), whereas monotherapy and combination therapy did not differ (*p* = 0.855). Multidrug resistance was linked to worse survival (log-rank *p* = 0.001). In multivariable models for 30-day mortality, only active treatment (aHR: 0.14; 95% CI: 0.02–0.88) and microbiological cure (aHR: 0.08; 95% CI: 0.01–0.47) remained independently protective. These data suggest that outcomes in SM-BSI are driven primarily by the receipt of appropriate therapy and achievement of microbiological clearance, reinforcing the need for prompt source control, optimized antimicrobial treatment, and continued development of novel therapeutic strategies to improve outcomes in this challenging infection.

## 1. Introduction

*Stenotrophomonas maltophilia* (SM) has emerged globally as a significant opportunistic, non-fermenting, Gram-negative pathogen responsible for a spectrum of healthcare-associated infections [1,2]. While once considered a bacterium of low virulence, it is now recognized as a leading cause of morbidity and mortality, particularly among critically ill, immunocompromised, and long-term hospitalized patients [3,4]. Bloodstream infections (BSIs) represent one of the most severe clinical manifestations and can significantly affect the pathogen’s intrinsic and acquired multidrug resistance (MDR) [5].

The incidence of SM infections has been steadily increasing, a trend attributed to the growing population of at-risk patients, the widespread use of broad-spectrum antibiotics such as carbapenems, and the increased utilization of invasive medical devices [6,7]. Established risk factors for acquiring an *S. maltophilia* BSI are well documented and include admission to an intensive care unit (ICU), prolonged hospitalization, mechanical ventilation, the presence of central venous catheters, underlying malignancies (particularly hematological ones), neutropenia, and recent surgical procedures [8,9].

The clinical management of *S. maltophilia* BSI is a formidable challenge. The bacterium exhibits intrinsic resistance to multiple antibiotic classes, including carbapenems, most cephalosporins, and aminoglycosides, primarily through the production of inducible beta-lactamases (L1 and L2) and multidrug efflux pumps [2,10]. Consequently, therapeutic options are severely limited. Trimethoprim–sulfamethoxazole (TMP-SMX) has historically been the treatment of choice; however, resistance rates are rising worldwide, further complicating therapeutic decisions [11].

Therefore, a substantial therapeutic uncertainty surrounds the optimal management of SM infections, both in the choice of active agents and in whether to use monotherapy or combinations. The recent European Committee on Antimicrobial Susceptibility Testing (EUCAST) guidance explicitly underscores the paucity of high-quality clinical data, notes that pharmacokinetic/pharmacodynamic (PK/PD) cut-offs for most agents sit below ECOFFs, and highlights unresolved questions on whether combination regimens are truly superior to monotherapy and which drugs should be prioritized [12]. In contrast, the 2024 IDSA AMR Guidance favors combination therapy, recommending the use of at least two active agents, such as cefiderocol, minocycline, TMP-SMX, or levofloxacin, at least until clinical improvement, while also cautioning that comparative clinical evidence remains limited and heterogeneous [13]. Overall, the absence of large, randomized trials and the inconsistent observational data contribute to a lack of consensus, reinforcing the need for timely active therapy guided by local susceptibility, patient factors, and evolving stewardship frameworks.

These uncertainties and the limited therapeutic arsenal contribute to the high mortality rates associated with SM-BSI, which have been reported to range from 20% to as high as 69% in different patient populations [9,14].

While the risk factors for the acquisition of *S. maltophilia* are well established, there is less clarity regarding the specific clinical and microbiological factors that independently predict mortality once an MDR BSI has developed. Differentiating between predictors of infection versus predictors of poor outcome is critical for improving patient management, guiding antimicrobial stewardship efforts, and developing targeted therapeutic strategies.

Therefore, the objective of this study was to identify the drivers of all-cause mortality in patients with MDR *Stenotrophomonas maltophilia* BSIs. By elucidating these key prognostic markers, we aim to provide clinicians with the evidence needed to better stratify patient risk, identify who may benefit from more aggressive or alternative treatment regimens, and ultimately improve clinical outcomes.

## 2. Materials and Methods

### 2.1. Study Design and Population

All patients who developed a microbiologically documented SM-BSI between September 2018 and December 2024 at the “Federico II” hospital, an 800-bed university hospital in Naples, Italy, were retrospectively recruited. Inclusion criteria were patients with one or more positive blood cultures for SM, age ≥ 18 years, and available clinical and laboratory follow-up data. The collection of the first-time positive blood culture was used to define the date of SM-BSI onset. An SM-BSI was defined as any SM isolation on blood culture in the event of fever or other clinical signs consistent with infection. In patients with multiple episodes of bacteremia caused by SM, only the first episode of infection was used for the analysis. An intra-ward cluster was defined as the detection of at least two SM-BSI cases in the same ward during a 14-day interval. A concomitant bloodstream infection was defined as bacteriemia due to pathogens other than SM that were detected within 3 days of the SM-BSI diagnosis. Microbiological or clinical SM-BSI cure was indicated by a test showing eradication of the infection and/or signs/symptoms, and laboratory findings suggestive of clinical resolution. MDR SM strains were defined as those with acquired non-susceptibility to at least one agent in three or more antimicrobial classes (cephalosporins, tigecycline, quinolones, and trimethoprim/sulfamethoxazole). Active antimicrobial therapy was defined as intravenous administration of at least one antibiotic demonstrating in vitro efficacy against the patient’s *S. maltophilia* isolate based on the susceptibility testing results described above. Therapy was considered active only if it was administered at standard or adjusted doses appropriate for the site of infection and the patient’s renal function. Empirical regimens started before susceptibility results were available were retrospectively classified as active only if they included an agent with documented in vitro activity against the isolate. Inadequate treatment was defined as (a) spectrum inadequacy (failure to receive any antimicrobial agent with in vitro activity against the isolated SM strain within 7 days of BSI onset) or (b) duration inadequacy (active therapy duration was less than 7 days in patients who survived beyond the treatment period). Patients meeting either criterion were classified as receiving inadequate treatment.

### 2.2. Data Collection

Data were collected from paper charts and electronic medical records. In particular, data regarding age, sex, hospitalization ward and eventual intra-hospital cluster, year of infection, comorbidities, the presence of risk factors for infection (i.e., length of hospitalization > 2 weeks, mechanical ventilation, central venous catheters, and immunosuppressive condition), clinical and laboratory parameters at infection (including white blood cell count, lymphocytic count, platelet count, serum creatinine, albumin, fibrinogen, c-reactive protein, procalcitonin, and concomitant sepsis or pneumonia), microbiologic results (including antimicrobial susceptibility profiles of the isolated SM and eventual concomitant infections) were reviewed. Moreover, the median follow-up time, the time between hospitalization and SM-BSI, the total length of hospital stay, the SM-BSI clinical/microbiological resolution at 14 days or last follow-up, the 14-day and 28-day mortality, and the time to SM-BSI and death were collected from the electronic hospital database. For patients who died before completing the intended treatment course, treatment duration was calculated as the actual number of days of active antimicrobial therapy received prior to death. Patients who died within 7 days of BSI onset without having received any active therapy were classified in the ‘<7 days’ category with zero days of active treatment. Patients who died within 48 h of BSI onset were excluded from the sensitivity analyses.

### 2.3. Microbiological Assays

Positive blood culture samples were detected using an automated blood culture system (BACTEC™, Becton Dickinson GmbH, Heidelberg, Germany). Positive samples were inoculated on multiple plate types (Becton Dickinson, Heidelberg, Germany) for 24 h at 36 °C. Conventional microbiological methods for Gram-negative bacilli using an automatic identification system (Phoenix 100^®^, NMIC/ID Becton Dickinson, Franklin Lakes, NJ, USA) and Matrix-Assisted Laser Desorption/Ionization–Time-of-Flight Mass Spectrometry (MALDI-TOF MS; Bruker, Bremen, Germany) were used to identify the culture isolate. The susceptibility of *S. maltophilia* isolates to antimicrobial agents were determined using an automatic system (Phoenix 100^®^, NMIC/ID Becton Dickinson, Franklin Lakes, NJ, USA). In addition, the susceptibility of the isolates was evaluated against cefiderocol using the agar disk diffusion method on Mueller–Hinton agar and a commercially available broth microdilution method (Sensititre cefiderocol minimum inhibitory concentration (MIC) panel CMP1SHIH; ThermoFisher, Waltham, MA, USA) according to the manufacturer’s instructions. When disk diffusion was used for screening (30 µg), isolates with zone diameters in the area of technical uncertainty were confirmed using the broth microdilution method.

Inhibition zone diameters and MIC values were interpreted and categorized according to the EUCAST 2024 species table for *S. maltophilia*, which currently only provides breakpoints for TMP–SMX (1:19; breakpoints expressed as the trimethoprim component). EUCAST lists S ≥ 50 mm/R < 16 mm for disk diffusion and emphasizes that growth may occur within inhibition zones [15]. For agents without EUCAST species breakpoints, we applied the CLSI M100-Ed34 (2024) criteria: cefiderocol susceptible at an MIC ≤ 4 μg/mL and levofloxacin susceptible at an MIC ≤ 2 μg/mL [16]. For tigecycline, MIC values were reported without categorical interpretation as neither EUCAST nor CLSI provide species-specific breakpoints for *S. maltophilia* [15,16,17,18,19,20]. Moreover, susceptibility to ceftazidime was evaluated (although CLSI SM breakpoints were recently removed); aztreonam–avibactam susceptibility was unavailable.

### 2.4. Statistical Analysis

The conformity of the obtained data to a normal distribution was examined using analytical methods (Kolmogorov–Smirnov test). In the reporting of the descriptive statistics of the study, for continuous numerical variables, mean ± standard deviation (SD) is given in the case of a Gaussian distribution and median and interquartile range (IQR) are given in the case of a non-Gaussian distribution; numbers and percentages are given for categorical variables. Comparisons of categorical variables were performed using Chi-square (χ^2^) or Fisher’s test (f). In order to compare continuous variables, an independent-sample *t*-test or Mann–Whitney U-test (U) was used. Kaplan–Meier survival analyses and log rank tests were used to compare the 30-day mortality rates. The association between the 30-day mortality and a variety of potential predictors was also investigated using a Cox regression analysis. All results were expressed as hazard ratios (HRs) with 95% confidence intervals (CIs). To evaluate the individual contribution of each independent factor, variables that showed a significant association in the univariate analysis were included in multivariate Cox regression model. For the variable selection strategy for multivariate analysis, given the limited sample size and number of outcome events (*n* = 16 deaths), we employed a conservative variable selection strategy to avoid model overfitting. Following the principle of approximately 10 events per predictor variable, we restricted the final multivariate Cox model to a maximum of two covariates. Variables were considered candidates for multivariate analysis if they achieved *p* < 0.05 in the univariate analysis. Among eligible candidates, we prioritized variables based on biological plausibility and clinical relevance to SM-BSI outcomes.

For all tests, *p* < 0.05 was chosen as the statistical significance limit value. The SPSS program, version 30.0.0 (IBM Corp., Armonk, NY, USA), was used in the analysis of the data.

### 2.5. Antimicrobial Dosing Regimens

- TMP-SMX: 15–20 mg/kg/day (trimethoprim component) in 3–4 IV doses; 50% reduction for CrCl < 30 mL/min.
- Levofloxacin: 750 mg once daily IV; 750 mg q48 h for CrCl < 50 mL/min.
- Tigecycline: 100 mg loading dose, then 50 mg q12 h; no renal adjustment.
- Cefiderocol: 2 g q8 h (3 h infusion); 1.5 g q8 h for CrCl 30–59; 1 g q8 h for CrCl 15–29 mL/min.

### 2.6. Use of Artificial Intelligence

Generative artificial intelligence (Claude, Anthropic, San Francisco, CA, USA) was used in English language editing, text revision, and figure generation. All AI-generated content was critically reviewed, verified, and edited by the authors who assume full responsibility for the final published work.

## 3. Results

### 3.1. Study Population and Patient Characteristics

During the study period, a total of 43 patients with a microbiologically documented SM-BSI were identified. The study population was predominantly male (61%, *n* = 26), with a median age of 63 years (IQR 55–73), and most patients (79%, *n* = 34) were older than 50 years. Patients were distributed across medical units (*n* = 22, 51%) and surgical units or ICUs (*n* = 21, 49%; 7 in a surgical unit and 14 in an ICU), with no significant demographic differences between these two groups in terms of age or sex (Table 1).

The cohort exhibited a substantial burden of comorbidities, with a median of three comorbidities per patient (IQR 2–4), and 68% (*n* = 28) presenting with more than two concurrent conditions. The most prevalent comorbidities were cardiovascular disease (61%, *n* = 25), onco-hematologic malignancies (51%, *n* = 22), diabetes mellitus (35%, *n* = 15), and chronic kidney disease (34%, *n* = 14). Notably, the prevalence of onco-hematologic disease differed significantly between ward types and was more common in medical units (73%) compared to surgical/ICU settings (29%, *p* = 0.004), while central nervous system disorders were more common in non-medical patients (43% vs. 15%, *p* = 0.050), reflecting the distinct patient populations served by these units (Table 1).

### 3.2. Risk Factors and Clinical Presentation

Several risk factors for SM-BSI were identified in the study population. A substantial proportion of patients (49%, *n* = 21) had been hospitalized for more than two weeks prior to SM-BSI onset (Table 1), especially in surgical/ICU settings (71% vs. 27%, *p* = 0.033). The presence of central venous catheters was nearly ubiquitous (86%, *n* = 31), with no significant difference between ward types. However, certain risk factors showed marked variation by setting: mechanical ventilation was exclusively observed in ICU patients (42%, *n* = 8, *p* = 0.004), while immunosuppressive conditions were predominantly found in medical unit patients (75% (*n* = 12) vs. 16% (*n* = 3), *p* < 0.001).

Prior antibiotic exposure was documented in 56% of patients (*n* = 24), with a significantly higher rate in surgical/ICU settings (81%) compared to medical units (32%, *p* = 0.004). Specifically, prior carbapenem exposure was observed in 35% of all patients (*n* = 15), with a trend toward higher prevalence in surgical/ICU patients (57% vs. 15%, *p* = 0.053). One-third of the cases (33%, *n* = 14) occurred as part of an intra-hospital cluster (Table 1).

At the time of infection, 42% of patients (*n* = 18) met the criteria for sepsis. The laboratory findings revealed significant hematological and biochemical abnormalities: 52% had white blood cell counts ≥ 6000 cells/μL (significantly higher in surgical/ICU patients, 81% vs. 25%, *p* < 0.001), 44% presented with thrombocytopenia (platelet count < 150,000 cells/μL), and 47% had hypoalbuminemia (<3 g/dL), which was more common in surgical/ICU settings (67% vs. 30%, *p* = 0.048). Concomitant infections were documented in 72% of patients (*n* = 31), with a significantly higher prevalence in surgical units/ICUs (99% vs. 50%, *p* = 0.003). Concomitant pneumonia was present in 40% of cases (*n* = 17), showing a marked difference between surgical/ICU (67%) and medical unit (15%) patients (*p* = 0.001) (Table 1).

### 3.3. Microbiological Findings and Antimicrobial Resistance Patterns

Analysis of antimicrobial susceptibility profiles revealed significant temporal changes in resistance patterns when comparing isolates from the early period (2018–2021, *n* = 19) to the more recent period (2022–2024, *n* = 24) (Table 2).

For TMP-SMX, the mean MIC was 0.04 mg/L (SD ± 0.02) but it notably decreased from 0.05 mg/L in 2018–2021 to 0.03 mg/L in 2022–2024 (*p* = 0.013) (Table 2). Despite this decrease in MIC values, a dramatic shift in susceptibility patterns was observed: while 53% of isolates were categorized as susceptible in 2018–2021, none of the isolates from 2022–2024 met the susceptibility criteria (*p* < 0.001). Instead, 100% of recent isolates fell into the “susceptible, increased exposure” category compared to only 37% in the earlier period (*p* < 0.001). Full resistance to TMP-SMX remained rare throughout the study period (2%, *n* = 1).

Levofloxacin resistance was prevalent throughout the study period, and identified in 86% of isolates (*n* = 37). A significant temporal change was observed, with all isolates from 2018–2021 being resistant (100%) compared to 75% in the 2022–2024 period (*p* = 0.027). The mean levofloxacin MIC was 1.44 mg/L (SD ± 0.68) (Table 2).

There were high rates of resistance to tigecycline (78% of isolates, *n* = 28). A marked temporal trend was evident, with universal resistance (100%) in 2018–2021 declining to 53% in 2022–2024 (*p* < 0.001). The mean tigecycline MIC decreased significantly from 2.0 mg/L in the early period to 1.35 mg/L in the recent period (*p* = 0.008) (Table 2).

Remarkably, no resistance to cefiderocol was detected in any isolate throughout the study period. The median disk diffusion diameter was 28 mm (IQR 24–32), with no significant differences between time periods.

Overall, 91% of isolates (*n* = 39) demonstrated resistance to at least two antimicrobial classes (Table 2). All isolates were resistant to ceftazidime. Critically, 61% of isolates (*n* = 26) met the criteria for multidrug resistance (MDR), which is defined as resistance to at least three antimicrobial classes. MDR prevalence showed a significant temporal decline, from 100% in 2018–2021 (combined resistance to ceftazidime, tigecycline, and levofloxacin) to 29% in 2022–2024 (*p* < 0.001).

The median time from hospitalization to SM-BSI onset was 18 days (IQR 5–29) (Table 2). The median number of positive blood cultures per patient was one (IQR 1–2), with 49% of patients (*n* = 17) having two or more positive blood cultures. Concomitant microbiologically documented *S. maltophilia* pneumonia was identified in 16% of cases (*n* = 6). Concomitant BSI due to other pathogens within 72 h occurred in 34% of patients (*n* = 12). COVID-19 co-infection was present in 9% of patients (*n* = 4), and fungal co-infections in 17% (*n* = 6).

### 3.4. Treatment Characteristics and Clinical Outcomes

Active antimicrobial therapy was administered to 74% of patients (*n* = 32), with no significant difference between ward types (Table 3).

TMP-SMX-based regimens were the most employed treatment strategy, which were used in 61% of cases (*n* = 26) (Table 3). Combination therapy was utilized in 23% of patients (*n* = 10). The median time from BSI onset to initiation of appropriate antibiotic therapy was 4 days (IQR 3–5), with 74% of patients (*n* = 34) receiving antibiotics more than 96 h after BSI detection. Treatment duration was highly variable, with a median of 7 days (IQR 0–12). A substantial proportion of patients (44%, *n* = 19) received therapy for less than 7 days, while only 21% (*n* = 9) received 14 days or more of treatment. Inadequate treatment, defined as spectrum or duration deficiencies, was documented in 46% of cases (*n* = 19) (Table 3).

Clinical outcomes at 14 days showed that 70% of patients (*n* = 30) achieved clinical resolution, while microbiological cure was documented in 61% of cases (*n* = 26) (Table 3). The overall 14-day mortality rate was 28% (*n* = 12). However, the 30-day mortality rate was substantial at 37% (*n* = 16), with no statistically significant difference between medical units (27%) and surgical/ICU settings (48%, *p* = 0.168). Among the patients who died, the median time to death was 10 days (IQR: 2–18). The median follow-up duration after BSI was 22 days (IQR: 7–30).

### 3.5. Predictive Factors for 30-Day Mortality

**Figure 1:**
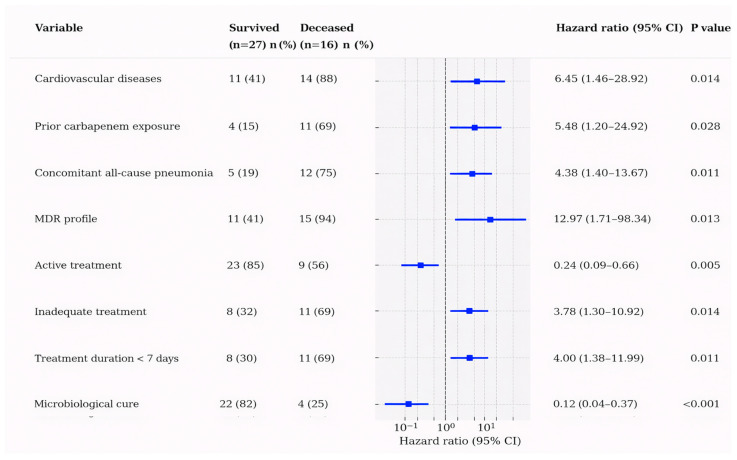
Univariate analysis of risk factors for mortality in *S. maltophilia* bloodstream infection.

Univariate Cox regression analysis identified multiple factors significantly associated with 30-day mortality (Figure 1).

Cardiovascular disease was present in 88% of deceased patients versus 41% of survivors (HR: 6.450; 95% CI: 1.458–28.92; *p* = 0.014) (Figure 1). Prior carbapenem exposure showed a strong association with mortality, affecting 69% of deceased patients compared to 15% of survivors (HR: 5.476; 95% CI: 1.203–24.92; *p* = 0.028). Concomitant all-cause pneumonia was significantly more prevalent among deceased patients (75% vs. 19%; HR: 4.382; 95% CI: 1.404–13.67; *p* = 0.011).

The MDR profile emerged as a critical predictor, with 94% of deceased patients having MDR isolates compared to 41% of survivors (HR: 12.97; 95% CI: 1.710–98.34; *p* = 0.013) (Figure 1). Treatment-related factors also demonstrated significant associations: active treatment was protective (HR: 0.241; 95% CI: 0.089–0.656; *p* = 0.005), while inadequate treatment (for spectrum or extent) was associated with increased mortality (HR: 3.776; 95% CI: 1.305–10.92; *p* = 0.014). A treatment duration of less than 7 days was significantly associated with mortality (HR: 3.996; 95% CI: 1.382–11.59; *p* = 0.011).

Most notably, microbiological eradication demonstrated the strongest protective effect: it occurred in 82% of survivors but only 25% of deceased patients (HR: 0.118; 95% CI: 0.038–0.373; *p* < 0.001) (Figure 1).

**Figure 2:**
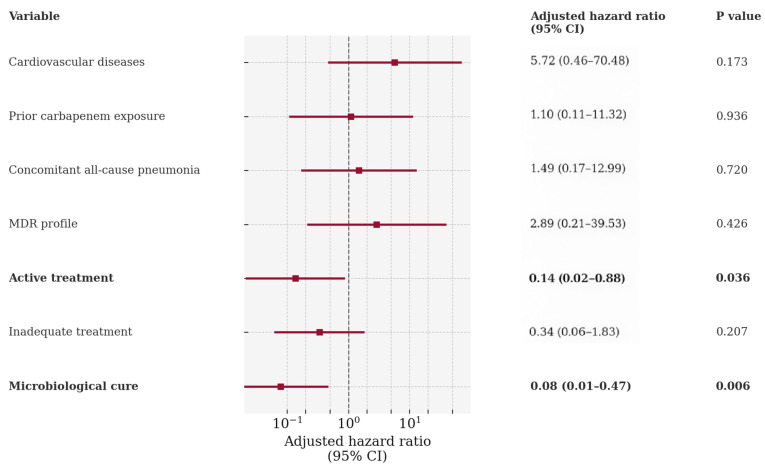
Independent predictors of 14-day mortality in *S. maltophilia* BSI (multivariable Cox model).

In the multivariate Cox regression analysis, only two factors retained independent significance (Figure 2).

Appropriate treatment remained a strong independent predictor of survival (adjusted HR: 0.137; 95% CI: 0.021–0.878; *p* = 0.036), while microbiological cure showed the most robust independent protective effect (adjusted HR: 0.078; 95% CI: 0.013–0.474; *p* = 0.006) (Figure 2). Other factors, including cardiovascular disease, prior carbapenem exposure, concomitant pneumonia, and MDR profile, while significant in the univariate analysis, did not maintain independent significance in the multivariate model. A treatment duration shorter than 7 days was excluded from the analysis because these cases were included in the “inadequate treatment” category.

**Figure 3:**
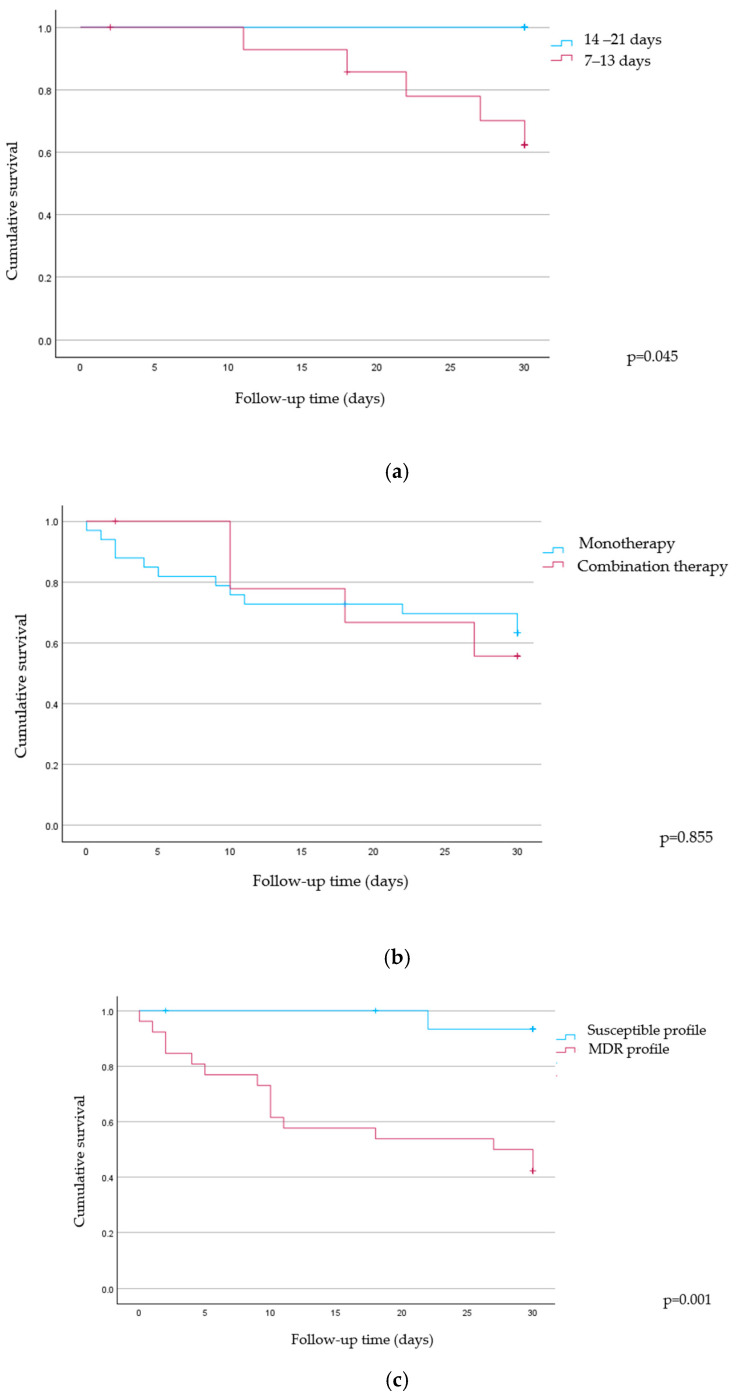
Survival during 30-day follow-up in *S. maltophilia* bloodstream infection: (**a**) antibiotic duration 14–21 vs. 7–13 days (*p* = 0.045); (**b**) mono vs. combination (*p* = 0.855); (**c**) susceptible vs. MDR (*p* = 0.001). Ticks = censored.

In time-to-event Kaplan–Meier analyses, antibiotic duration was associated with 30-day survival. There were no deaths among the patients treated for 14–21 days at follow-up (0/9), whereas there were 5/15 deaths among those treated for 7–13 days. The survival curves differed according to the log-rank test (χ^2^ = 4.03, *p* = 0.045) (Figure 3a). By contrast, treatment regimen did not influence survival: monotherapy (12/33 deaths; mean survival: 22.9 days; SE: 2.06) and combination therapy (4/10 deaths; mean survival: 23.9 days; SE: 2.76) did not show a significant difference (*p* = 0.855) (Figure 3b). Finally, resistance profile strongly affected outcomes: patients with isolates resistant to ≥3 classes (MDR) showed shorter survival (mean: 19.2 days; SE: 2.43; median: ~27 days) compared with those with a susceptible profile (mean: 29.5 days; SE: 0.52), with a clear separation of curves (χ^2^ = 10.48, *p* = 0.001) (Figure 3c).

## 4. Discussion

This retrospective study provides comprehensive insights into the clinical characteristics, antimicrobial resistance patterns, and mortality predictors of *Stenotrophomonas maltophilia* bloodstream infections over a six-year period at a large Italian university hospital. Our findings reveal several important observations that contribute to our understanding of this challenging nosocomial pathogen and have direct implications for clinical management.

The 30-day mortality rate of 37% in our cohort is consistent with the contemporary literature: 37.4% in elderly patients [21], 40.5% in a meta-analysis of 1248 patients [22], 41.6% in a 12-year surveillance study [23], and 46.1% in a recent Turkish multicenter study [24]. ICU-specific cohorts report a mortality rate approaching 50% without appropriate therapy [24,25]. Our intermediate rate likely reflects the mixed patient population and the 74% who received active treatment.

In terms of prognostic factors, our multivariate analysis identified two independent predictors of survival: appropriate antimicrobial treatment (adjusted HR: 0.137; 95% CI: 0.021–0.878; *p* = 0.036) and microbiological cure (adjusted HR: 0.078; 95% CI: 0.013–0.474; *p* = 0.006). Most compellingly, Lai et al. reported a dramatic reduction in 14-day mortality among ICU patients receiving appropriate therapy compared to inappropriate therapy (10.5% vs. 46.9%, *p* < 0.001) [25], with this difference persisting even after propensity score matching (11.5% vs. 39.3%, *p* < 0.001). Gezer et al. similarly reported appropriate therapy as independently protective (HR: 0.35) [24].

The protective effect of microbiological cure observed in our study aligns with the established principles of infectious disease management. Mojica et al. highlighted in their comprehensive review that microbiological eradication with active therapy is associated with a decreased death risk in SM infections [6]. The median 4-day delay (IQR 3–5) from BSI to appropriate therapy reflects diagnostic constraints: blood culture positivity (24–48 h), species identification (24 h), and susceptibility testing (48–72 h). Consequently, 74% of patients received therapy >96 h after BSI detection. While time-to-treatment did not achieve independent significance in the multivariate analysis possibly due to its narrow IQR and collinearity with MDR status, Lai et al. demonstrated that early appropriate therapy significantly improves ICU outcomes [25]. These findings underscore the need for rapid molecular diagnostics and, in high-risk patients with SM colonization or prolonged ICU stay, consideration of empirical SM-active coverage pending culture results.

Furthermore, recent clinical experience with novel agents has reinforced this concept: in a case series of eight BSI patients treated with cefiderocol, microbiological clearance was achieved in the majority of patients with follow-up cultures, and notably, no increase in cefiderocol MICs was observed during treatment [26]. This contrasts sharply with respiratory infections, where resistance development during therapy has been documented, suggesting that BSI may be more amenable to definitive microbiological cure.

Interestingly, while several factors showed significant associations with mortality in the univariate analysis including cardiovascular disease (HR: 6.450), MDR profile (HR: 12.97), concomitant pneumonia (HR: 4.382), and prior carbapenem exposure (HR: 5.476), these factors did not maintain independent significance in our multivariate model. This may reflect the overriding importance of appropriate treatment and microbiological cure, which can potentially mitigate the adverse effects of these risk factors when achieved. However, these univariate associations remain clinically meaningful. The strong association of cardiovascular disease with mortality in our cohort is consistent with meta-analytic evidence identifying chronic kidney disease and other comorbidities as significant risk factors [22]. The relationship between prior carbapenem exposure and mortality has been particularly well documented: in a recent study of 140 adult hematological patients with a 28-day mortality rate of 31.43%, 69.29% developed breakthrough SM bacteremia during active carbapenem therapy [27]. Similarly, Koh et al. reported that prior carbapenem exposure was significantly higher in non-survivors compared to survivors (81.7% vs. 53.0%, *p* < 0.001) [23]. Several univariate predictors (cardiovascular disease (HR: 6.45), MDR (HR: 12.97), pneumonia (HR: 4.38), and carbapenem exposure (HR: 5.48)) lost multivariate significance. However, this loss of significance does not diminish their clinical relevance; these factors are still high-risk markers warranting early ID consultation, empirical SM coverage, and aggressive source control. It is alto to be noted that the limited sample size (*n* = 43) may have reduced the statistical power.

One of the most striking findings of our study is the significant temporal shift in antimicrobial resistance patterns, with MDR prevalence declining from 100% in 2018–2021 to 29% in 2022–2024 (*p* < 0.001). This encouraging trend contrasts with the general perception of inexorably increasing antimicrobial resistance and deserves careful interpretation. Our observation is supported by recent surveillance data demonstrating the dynamic nature of *S. maltophilia* resistance patterns. AlFonaisan et al., in a comprehensive 19-year retrospective analysis of 4466 patients, documented progressive but fluctuating resistance trends, with TMP-SMX susceptibility declining from 94% to 80% over the study period yet maintaining an average susceptibility of 85.7% [28]. Similarly, Gezer et al. observed temporal changes in TMP-SMX resistance, which increased from 1.7% in 2018–2020 to 10.4% in 2021–2023 (*p* = 0.033) [24].

The decline in MDR rates observed in our cohort may reflect several factors, including improved antimicrobial stewardship practices (including ad hoc programs for the ICU of our hospital since 2021), enhanced infection control measures, changes in empirical antibiotic selection pressures, or epidemiological shifts in circulating strain populations. The substantial decrease in tigecycline resistance from universal resistance (100%) in 2018–2021 to 53% in 2022–2024 (*p* < 0.001), with corresponding decreases in mean MIC values, suggests genuine improvement rather than merely definitional changes. This trend parallels global observations, though some regions have reported contradictory patterns. Notably, a global meta-analysis documented that tigecycline resistance increased four-fold after 2010 (from 8.2% to 30.2%) [29], highlighting substantial geographic and temporal heterogeneity. There are several possible explanations for this trend: (1) antimicrobial stewardship targeting carbapenem prescription (ICU program started in 2021); (2) reduced upfront carbapenem use in empirical protocols; (3) enhanced infection prevention during COVID-19; or (4) clonal replacement requiring WGS for confirmation. The universal cefiderocol susceptibility (MIC90 0.12 μg/mL) aligns with global data (4% resistance) [29], reflecting siderophore-mediated uptake bypassing conventional resistance. Sporadic resistance emergence in respiratory infections warrants surveillance [26].

The shift in TMP-SMX categorization, from 53% susceptible (2018–2021) to 100%, “susceptible, increased exposure” (2022–2024) occurred despite decreasing MICs (0.05 to 0.03 mg/L, *p* = 0.013), suggesting revised interpretive criteria rather than true resistance acquisition. Global TMP-SMX resistance ranges from 9.2% to 14.7% [11,29]. While meta-analysis data suggest higher mortality with TMP-SMX resistance versus fluoroquinolone resistance (OR: 1.46) [30], confounding limits the interpretation; both remain viable first-line options when susceptible.

The universal susceptibility to cefiderocol observed throughout our study period represents a particularly encouraging finding. This is strongly corroborated by recent evidence: in vitro studies of 37 MDR *S. maltophilia* isolates (resistant to levofloxacin and/or TMP-SMX) demonstrated 100% susceptibility to cefiderocol at the CLSI breakpoint (≤4 μg/mL) with an MIC90 of 0.5 μg/mL [31]. Global surveillance has confirmed cefiderocol’s exceptional activity, with only 4% resistance reported worldwide—the second-lowest rate after minocycline (3%) [29].

When analyzing risk factors and patient populations at risk, the high prevalence of onco-hematologic malignancies in our cohort (51% overall, 73% in medical units) aligns with well-documented associations between immunosuppression and SM infections. This population represents a particularly vulnerable group requiring heightened surveillance and consideration of SM-directed empirical therapy in appropriate clinical contexts.

The significance of concomitant pneumonia as a mortality predictor (75% in deceased patients vs. 19% in survivors; HR: 4.382 in univariate analysis) reflects both the severity of polymicrobial or multi-site infections and the specific challenges of treating SM pneumonia. Pharmacokinetic/pharmacodynamic considerations suggest that achieving adequate drug exposure at pulmonary sites may be particularly challenging. Mojica et al. noted that levofloxacin may offer superior outcomes for pneumonia due to favorable lung penetration, while BSI may be more responsive to a broader range of agents [6].

The role of central venous catheters, present in 86% of our patients, warrants specific discussion. While the univariate analysis did not identify CVC presence as a mortality predictor in our cohort, likely due to its near-universal presence, catheter removal has been identified as an independent protective factor in other studies (HR = 0.31; 95% CI: 0.16–0.60; *p* = 0.001) [24]. This suggests that when feasible, source control through CVC removal should be a priority in management, particularly for catheter-associated BSI.

Regarding treatment choices and antimicrobial stewardship implications, the finding that the median time from BSI onset to appropriate antibiotic initiation of 4 days in our cohort, with 74% of patients receiving antibiotics more than 96 h after BSI detection, highlights significant delays that may contribute to mortality. These delays reflect the challenges of timely pathogen identification, susceptibility testing, and recognition of SM as a true pathogen rather than a contaminant or colonizer.

The predominant use of TMP-SMX-based regimens (61% in our cohort) is consistent with guideline recommendations when susceptibility is confirmed. The role of combination therapy, however, remains controversial. Although 23% of our patients received combination therapy, we found no survival advantage over monotherapy after adjustment, whereas receipt of active therapy and achievement of microbiological clearance were the key determinants of the desired outcomes; moreover, a 14–21-day course appeared superior to a 7–13-day course for 30-day survival. These real-world signals help explain the persistent gap between guidelines and practice (e.g., 76.6% monotherapy in a 64-patient multicenter series despite IDSA guidance favoring combinations for severe disease [32]), likely reflecting both limited high-quality evidence and confounding by indication. Importantly, a recent study of deep-seated, monomicrobial *S. maltophilia* infections suggested that levofloxacin-based doublets (levofloxacin–minocycline or levofloxacin–TMP-SMX) may reduce clinical failure in high-inoculum/biofilm contexts, supporting a selective, phenotype-driven use of combinations rather than routine deployment in uncomplicated BSI [33]. In vitro synergy between cefiderocol and levofloxacin, minocycline, or TMP-SMX (44–67%) [31] is interesting but whether such interactions translate into superior clinical outcomes likely depends on source control, timely initiation of an active agent, and infected compartment. Altogether, our data and the external evidence point toward prioritizing early active therapy and microbiological clearance for SM BSI, reserving combination regimens for patients with deep-seated or otherwise high-risk infections while we await prospective trials to define their incremental benefit.

The inadequate treatment rate of 46% in our cohort, defined as spectrum or duration deficiencies, represents a significant opportunity for intervention. Treatment duration was highly variable (median 7 days, IQR 0–12), with 44% receiving less than 7 days of therapy. Shorter treatment duration (<7 days) was significantly associated with mortality in the univariate analysis (HR: 3.996; *p* = 0.011), though this association did not persist in the multivariate modeling. The optimal duration of therapy for SM-BSI remains undefined, and further research is needed to establish evidence-based treatment duration recommendations.

Moreover, our Kaplan–Meier analyses suggest that longer antibiotic courses (14–21 days) are associated with better 30-day survival, while combination therapy offers no observable advantage over monotherapy in this cohort. This finding likely reflects the clinical complexity of our cohort, which included a high proportion of ICU and immunocompromised patients, often with catheter-related infections and/or additional infectious foci, contexts where the “shorter is better” principle may not be applicable. Moreover, the link between MDR phenotype and substantially worse survival reinforces the clinical importance of early, active therapy and source control when resistance limits options. Interpretation should remain cautious: the 14–21-day group is small and event-free, raising concerns for confounding by indication and immortal-time bias (patients who die early cannot complete longer courses). Residual confounding (e.g., illness severity, source control, and comorbidities) may also influence these unadjusted comparisons. Nonetheless, the magnitude and consistency of the MDR effect, together with the neutral finding for combination therapy, align with clinical expectations and support focusing on timely, effective monotherapy when adequate activity is assured, reserving combinations for specific indications rather than survival benefit per se.

Finally, our findings have several immediate clinical implications. First, they reinforce the critical importance of appropriate antimicrobial therapy and microbiological cure as the primary modifiable factors influencing survival in SM-BSI. Clinicians should prioritize rapid pathogen identification, timely susceptibility testing, and prompt de-escalation or escalation to active agents. Second, the encouraging decline in MDR rates in our setting suggests that enhanced antimicrobial stewardship and infection control measures may be effective, though continued surveillance is essential to detect and respond to emerging resistance patterns. Third, the universal susceptibility to cefiderocol provides reassurance that therapeutic options exist even for highly resistant isolates, though clinical experience with this agent remains limited and careful patient selection is warranted.

The high mortality rates that persist despite modern therapy highlight the need for novel approaches beyond conventional antimicrobial treatment. These might include optimization of pharmacokinetic/pharmacodynamic exposure through therapeutic drug monitoring and aggressive source control measures. The development of rapid diagnostic techniques that can identify SM and predict antimicrobial susceptibility within hours rather than days represents a critical unmet need that could enable earlier appropriate therapy.

Future research should focus on several key areas. First, prospective, adequately powered studies comparing monotherapy versus combination therapy for SM-BSI are urgently needed to inform guideline recommendations. Second, the optimal treatment duration and dosing strategies, particularly for the “susceptible, increased exposure” category of TMP-SMX susceptibility, require systematic investigation. Third, the role of novel agents such as cefiderocol, aztreonam–avibactam, and optimized formulations of established agents need to be defined through comparative effectiveness studies. Fourth, the development and validation of prognostic models that can identify high-risk patients early in their clinical course could enable risk-stratified interventions. Finally, investigation of the molecular mechanisms underlying the temporal changes in resistance patterns observed in our study and others could provide insights into effective antimicrobial stewardship strategies.

Several limitations warrant consideration. First, the retrospective single-center design limits generalizability and precludes causal inference. Treatment-selection bias may have influenced findings: patients with better prognosis may have been more likely to receive and tolerate prolonged antibiotic courses, while clinicians may have withheld aggressive therapy from moribund patients, creating confounding by indication that could exaggerate the apparent protective effect of active treatment.

Second, antimicrobial exposure documentation accuracy cannot be verified retrospectively. Undocumented dose modifications, missed administrations, or recording errors may have led to misclassification of treatment adequacy. Timing of antibiotic initiation was based on pharmacy and nursing records, which may not precisely reflect actual administration.

Third, source control interventions were inconsistently documented. The time of central venous catheter removal, a critical intervention in catheter-related SM-BSI, was not systematically recorded, precluding analysis of source control timing impact on outcomes.

Fourth, the modest sample size (*n* = 43) the limited statistical power of the multivariate analysis and precluded subgroup analyses that might have identified differential treatment effects across patient populations.

Fifth, the resistance patterns and treatment practices at our institution may differ from those of other centers; the declining MDR prevalence observed may reflect local factors and requires multicenter validation.

Finally, the absence of whole-genome sequencing data prevented assessment of whether the temporal resistance changes reflected clonal replacement or within-strain resistance loss.

## 5. Conclusions

This study identified several key clinical findings in SM-BSI management. First, 30-day mortality remains substantial at 37% despite contemporary treatment approaches. Second, active antimicrobial therapy (aHR: 0.14; 95% CI: 0.03–0.70) and microbiological eradication (aHR: 0.08; 95% CI: 0.01–0.44) emerged as the only independent predictors of survival, emphasizing treatment adequacy over empirical risk stratification. Third, MDR prevalence declined markedly from 100% (2018–2021) to 29% (2022–2024), potentially reflecting an antimicrobial stewardship impact. Fourth, universal cefiderocol susceptibility (MIC90: 0.12 μg/mL) positions this agent as a reliable therapeutic option. Finally, the median 4-day delay to appropriate therapy highlights the urgent need for rapid diagnostic implementation in high-risk patients. The risk factors of cardiovascular disease, prior carbapenem exposure, concomitant pneumonia, and MDR profile, while not independently predictive in the multivariate analysis, remain clinically important for risk stratification and treatment planning. These findings emphasize the need for timely source control, optimized antimicrobial therapy, and continued development of novel therapeutic strategies to improve outcomes in this challenging infection. Future research should focus on prospective comparative effectiveness studies of treatment regimens, optimization of pharmacokinetic/pharmacodynamic exposure, and development of rapid diagnostic tools to enable earlier appropriate therapy.

## Figures and Tables

**Table 1:** Demographics, comorbidities, risk factors, and severity of patients with an SM-BSI stratified by ward.

		Ward		
	Total (*n* = 43) *n* (%)	Medical Units (*n* = 22) *n* (%)	Surgical/Intensive Care Units (*n* = 21) *n* (%)	*p* (χ^2^ Test)
**Male sex**	26 (61)	9 (41)	13 (62)	0.850
**Median age [years, IQR]**	63 [55–73]	64 [58–69]	60 [40–79]	0.903 (U)
Age > 50 years	34 (79)	19 (86)	15 (71)	0.281 (f)
**Comorbidities [median, IQR]**	3 [2–4]	3 [2–4]	4 [2–4]	0.511 (U)
Chronic kidney disease	14 (34)	5 (25)	9 (43)	0.228
Chronic liver disease	11 (27)	6 (30)	5 (24)	0.655
Cardiovascular disease	25 (61)	11 (55)	14 (67)	0.444
Diabetes	15 (35)	8 (40)	7 (33)	0.658
Onco-hematologic disease	22 (51)	16 (73)	6 (29)	**0.004**
Central nervous system disorders	12 (29)	3 (15)	9 (43)	**0.050**
More than 2 comorbidities	28 (68)	13 (65)	15 (71)	0.658
**Year of infection**				
2018	5 (12)	4 (18)	1 (5)	0.187 (f)
2019	5 (12)	4 (18)	1 (5)	0.179
2020	4 (10)	2 (9)	2 (10)	1.000 (f)
2021	5 (12)	0 (0)	5 (24)	**0.021 (f)**
2022	9 (21)	2 (9)	7 (33)	0.069 (f)
2023	8 (19)	4 (18)	4 (19)	1.000 (f)
2024	7 (16)	6 (27)	1 (5)	0.095 (f)
Years 2018–2021	19 (44)	10 (45)	9 (43)	0.864
Years 2022–2024	24 (56)	12 (55)	12 (57)	-
**Risk factors for infection**				
Length of hospitalization > 2 weeks	21 (49)	6 (27)	15 (71)	**0.033**
Mechanical ventilation	8 (23)	0 (0)	8 (42)	**0.004 (f)**
Central venous catheters	31 (86)	14 (82)	17 (89)	0.650 (f)
Immunosuppressive condition	15 (35)	12 (75)	3 (16)	**<0.001**
Intra-hospital cluster	14 (33)	8 (36)	6 (29)	**0.586**
Prior antibiotic exposure	24 (56)	7 (32)	17 (81)	**0.004**
Prior carbapenem exposure	15 (35)	3 (15)	12 (57)	0.053
**Severity scores at infection**				
Sepsis	18 (42)	10 (45)	8 (38)	0.229
WBC ≥ 6000 cells/uL	22 (52)	5 (25)	17 (81)	**<0.001**
Platelet count < 150.000 cells/uL	19 (44)	8 (40)	11 (52)	0.640
Albuminemia < 3 g/dL	20 (47)	6 (30)	14 (67)	**0.048**
eGFR < 70 mL/min/m^2^	13 (30)	3 (15)	10 (48)	0.052
Concomitant infections (overall)	31 (72)	11(50)	20 (99)	**0.003**
Concomitant pneumonia	17 (40)	3 (15)	14 (67)	**0.001**

**Table 2:** Temporal trends in antimicrobial susceptibility and resistance profiles in SM-BSI cases (2018–2021 vs. 2022–2024).

		Years		
	Total (*n* = 43) *n* (%)	2018–2021 (*n* = 19) *n* (%)	2022–2024 (*n* = 24) *n* (%)	*p* (χ^2^ Test)
**TMP-SMX susceptibility**				
MIC [mean, ±SD]	0.04 [±0.02]	0.05 [±0.01]	0.03 [±0.02]	**0.013 (U)**
Susceptible	10 (23)	10 (53)	0 (0)	**<0.001 (f)**
Susceptible, increased exposure	31 (72)	7 (37)	24 (100)	**<0.001**
Resistant	1 (2)	1 (5)	0 (0)	0.442 (f)
**Levofloxacin susceptibility**				
MIC [mean, ±SD]	1.44 [±0.68]	n/a	1.44 [±0.68]	-
Resistant	37 (86)	19 (100)	18 (75)	**0.027 (f)**
**Tigecycline susceptibility**				
MIC [mean, ±SD]	1.78 [±0.45]	2 [±0.00]	1.35 [±0.58]	**0.008 (U)**
Resistant	28 (78)	19 (100)	9 (53)	**<0.001 (f)**
**Cefiderocol susceptibility**				
MIC [mean, ±SD]	0.08 [0.03–0.12]	0.06 [0.06–0.06]	0.08 [0.03–0.12]	0.727
Disk diffusion diameter [median, IQR]	28 [24–32]	29 [25–32]	28 [24–32]	0.560 (U)
**Resistance profile**				
Resistance to at least 2 classes	39 (91)	19 (100)	20 (83)	0.118 (f)
Resistance to at least 3 classes (MDR)	26 (61)	19 (100)	7 (29)	**<0.001**
**Median time from hospitalization to SM-BSI [days, IQR]**	18 [5–29]	23 [6–31]	15 [3–26]	0.145
**Median number of positive blood cultures for SM [n, IQR]**	1 [1–2]	2 [1–3]	1 [1–2]	0.044 (U)
Two or more positive blood cultures for SM	17 (49)	12 (63)	5 (31)	0.060
**Concomitant microbiologically documented SM pneumonia**	6 (16)	2 (11)	4 (21)	0.660 (f)
**Concomitant BSI** **(± 72 h)**	12 (34)	7 (37)	5 (31)	0.728
**Concomitant COVID-19**	4 (9)	3 (16)	1 (4)	0.306 (f)
**Fungal coinfection**	6 (17)	4 (21)	2 (13)	0.666 (f)

**Table 3:** Ward-stratified treatment patterns and outcomes of *S. maltophilia* bloodstream infection.

		Ward		
	Total (*n* = 43) * n * (%)	Medical Units (*n* = 22) * n * (%)	Surgical/Intensive Care Units (*n* = 21) * n * (%)	* p *
**SM Treatment**				
Active therapy	32 (74)	17 (77)	15 (71)	0.661
Combination therapy	10 (23)	4 (18)	6 (29)	0.328 (f)
TMP-SMX-based treatment	26 (61)	13 (60)	13 (62)	0.659
Time from BSI to antibiotic start	4 [3–5]	4 [3–5]	4 [4–5]	0.913 (U)
More than 96 h from BSI to antibiotic start	34 (74)	15 (68)	19 (91)	0.132 (f)
Treatment duration (days)	7 [0–12]	7 [3–12]	7 [0–13]	0.980
<7 days	19 (44)	9 (41)	10 (48)	0.658
7–13 days	15 (35)	9 (41)	6 (29)	0.396
14 days or more	9 (21)	4 (18)	5 (24)	0.721 (f)
Inadequate treatment *	19 (46)	9 (45)	10 (48)	0.867
** Outcome at +14 days **				
Clinical resolution **	30 (70)	16 (73)	14 (67)	0.665
Microbiological resolution **	26 (61)	13 (60)	13 (62)	0.850
Mortality	12 (28)	5 (23)	7 (33)	0.438
** Mortality at +30 days **	16 (37)	6 (27)	10 (48)	0.168
Median time to death [days, IQR]	10 [2–18]	10 [4–13]	8 [2–21]	0.892 (U)
** Median follow-up after BSI [days, IQR] **	22 [7–30]	18 [9–30]	25 [3–30]	0.842 (U)

* Due to spectrum or duration. ** Or at last follow-up if the patient was deceased.

## Data Availability

The original contributions presented in this study are included in the article. Further inquiries can be directed to the corresponding authors.

## Abbreviations

The following abbreviations are used in this manuscript:

SM	*Stenotrophomonas maltophilia*
BSIs	blood stream infections
MDR	multidrug resistance
ICU	intensive care unit
TMP-SMX	trimethoprim–sulfamethoxazole
EUCAST	European Committee on Antimicrobial Susceptibility Testing
ECOFFs	EUCAST epidemiological cut-off values
IDSA	Infectious Diseases Society of America
AMR	antimicrobial resistance
MALDI-TOF	Matrix-Assisted Laser Desorption/Ionization–Time-of-Flight Mass Spectrometry
MIC	minimum inhibitory concentration
CLSI	Clinical & Laboratory Standards Institute
SD	standard deviation
IQR	interquartile range
HR	hazard ratio
CI	confidence interval
n/a	not available
SE	standard error
